# Progress in biological and medical research in the deep underground: an update

**DOI:** 10.3389/fpubh.2023.1249742

**Published:** 2023-08-10

**Authors:** Yuhao Zou, Ling Wang, Jirui Wen, Juan Cheng, Can Li, Zhizhen Hao, Jian Zou, Mingzhong Gao, Weimin Li, Jiang Wu, Heping Xie, Jifeng Liu

**Affiliations:** ^1^Department of Otolaryngology Head and Neck Surgery, West China Hospital, Sichuan University, Chengdu, China; ^2^Deep Underground Space Medical Center, West China Hospital, Sichuan University, Chengdu, China; ^3^College of Water Resources and Hydropower, Sichuan University, Chengdu, China; ^4^Institute of Deep Earth Science and Green Energy, Shenzhen University, Shenzhen, China; ^5^West China Hospital, Sichuan University, Chengdu, China

**Keywords:** deep underground laboratories, deep underground medicine, background radiation, cell growth, phenotypic changes

## Abstract

As the growing population of individuals residing or working in deep underground spaces for prolonged periods, it has become imperative to understand the influence of factors in the deep underground environment (DUGE) on living systems. Heping Xie has conceptualized the concept of deep underground medicine to identify factors in the DUGE that can have either detrimental or beneficial effects on human health. Over the past few years, an increasing number of studies have explored the molecular mechanisms that underlie the biological impacts of factors in the DUGE on model organisms and humans. Here, we present a summary of the present landscape of biological and medical research conducted in deep underground laboratories and propose promising avenues for future investigations in this field. Most research demonstrates that low background radiation can trigger a stress response and affect the growth, organelles, oxidative stress, defense capacity, and metabolism of cells. Studies show that residing and/or working in the DUGE has detrimental effects on human health. Employees working in deep mines suffer from intense discomfort caused by high temperature and humidity, which increase with depth, and experience fatigue and sleep disturbance. The negative impacts of the DUGE on human health may be induced by changes in the metabolism of specific amino acids; however, the cellular pathways remain to be elucidated. Biological and medical research must continue in deep underground laboratories and mines to guarantee the safe probing of uncharted depths as humans utilize the deep underground space.

## 1. Introduction

As resources in the earth’s superficial layers become scarce, many counties have initiated efforts to explore and exploit the deep-underground space (1, 2). Deep coal mining has occurred in Poland, Germany, the United Kingdom, Japan, and France since the 1980s, and currently, 55 coal mines have reached depths of over 1,000 m in China (3, 4).

Gaining insights into the effects of deep underground environment (DUGE) factors on living systems is essential for understanding the nature of the deep underground space and implementing measures to protect the health of the humans that use the deep underground space (5). In 2018, Heping Xie advocated the need for deep underground medicine (DUGM), a multidisciplinary approach to exploring the physiological, pathological and psychological impacts of factors in the DUGE on humans (5). DUGM will identify detrimental factors in the DUGE and facilitate the development of efficient and secure methods to harness factors that are beneficial for life.

**Figure 1:**
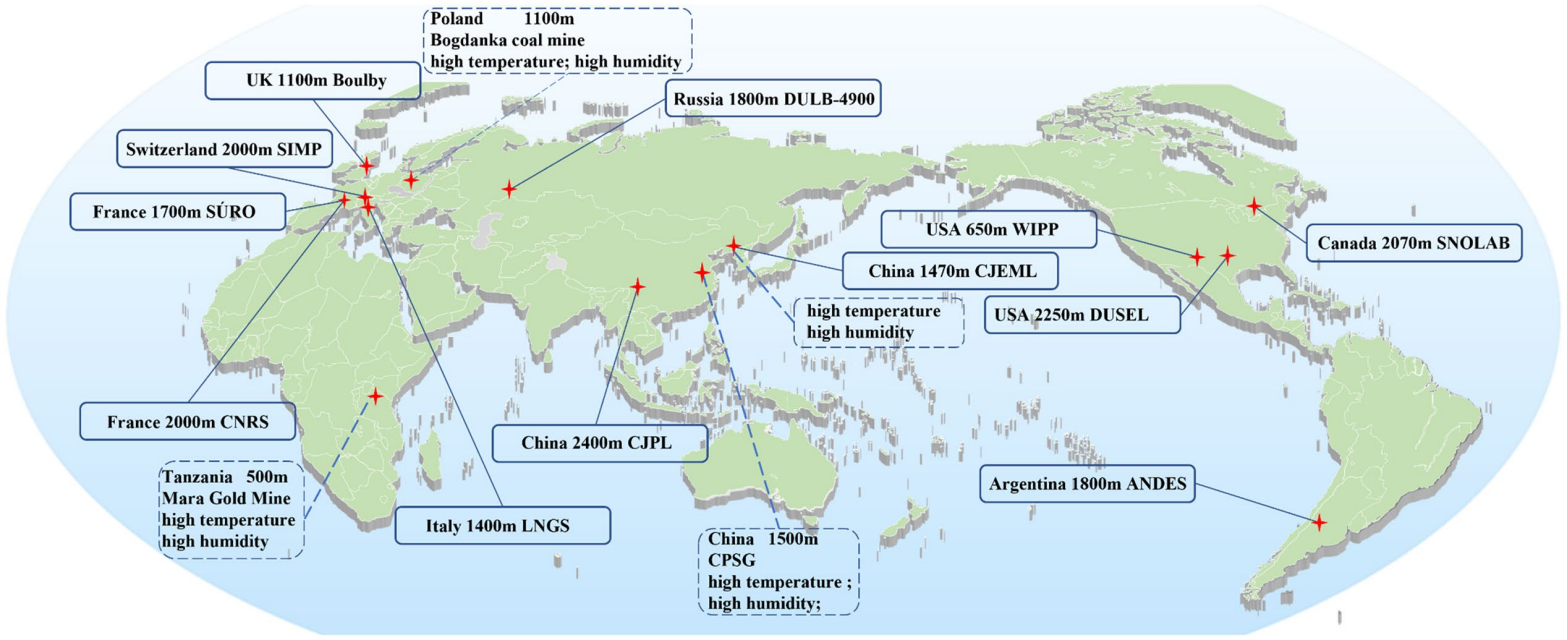
The location of DUGLs conducting biological research. SIMP, Simplon pass; WIPP, waste isolation pilot plant; CJEML, Laboratory of Jiapigou Minerals Limited Corporation of China National Gold Group Corporation; CJPL, Chinese Jinping Deep Underground Laboratory; DUSEL, Dusseldorf Underground Laboratory, United States; LNGS, the Gran Sasso National Laboratory; DULB-4900, the Baksan Neutrino Observatory; CPSG, China Pingmei Shenma Group; CNRS, Center National de la Recherche Scientifique; ANDES, Argentina Agua Negra Extremely Deep Underground Laboratory; and SNOLAB, Sudbury Neutrino Observatory Laboratory.

Research on factors in the DUGE is conducted in deep underground laboratories (DULs). Cosmic ray is reduced in DULs created under a rock overburden exceeding 1,000 m of water equivalent, creating a unique environment that allows investigations into rare occurrences in the fields of astroparticle physics and neutrino physics (6, 7). More recently, scientists have developed an interest in the biological impacts of low background radiation (LBR) on living organisms nurtured in DULs (6, 8). Currently, 12 DULs worldwide are engaged in biological research to elucidate the influence of factors in the DUGE on living systems (Figure 1).

Multiple experiments in DULs have revealed the notable detrimental impact of LBR in single-celled organisms (9). Since Prof. Xie proposed the concept of DUGM, an increasing number of DULs have extended their studies to multicellular organisms and started to explore the intricate molecular mechanisms that underlie the biological impacts of LBR (10, 11). Other studies are focused on the impact of factors in the DUGE on human physiological and psychological health. Here, we present a summary of the current landscape of biological and medical research in DULs and propose promising avenues for future investigations in this field.

## 2. Characteristics of the DUGE

To effectively comprehend the biological impacts of DUGE on living organisms, it is necessary to describe the various environmental factors present, including radiation, temperature, humidity, and air pressure. The total background radiation level in DULs is 5–10 times lower than above-ground laboratories (Table 1). Natural background radiation comprises γ radiation, neutrons, muons, and radon gas. In DULs, γ radiation originates from nuclear decays of ^222^Rn and ^220^Rn and their daughter products in the rocks and atmosphere (21); neutron flux is usually low, resulting from the decay of uranium and thorium in rocks and building materials (22); muons are produced by the interaction of cosmic radiation with the Earth’s atmosphere, with the rocky cover of the DULs providing effective shielding (23).

**Table 1:** Components of background radiation in deep underground laboratories.

Laboratory name	Depth	γ flux	Muon flux (cm^−2^ s^−1^)	Neutron flux (cm^−2^ s^−1^)	Radon (Bq m^−3^)	Reference
WIPP	650 m	8.27 * 10^−9^Gy/h	4.77 * 10^−7^	6 * 10^−8^	7	(12)
Boulby	1,100 m	0.128 (cm^–2^ s^–1^)	4.5 * 10^−8^	1.7 * 10^−6^ (E. 0.5 MeV)	2.4	(13, 14)
SNOLAB	2,000 m	8.2 * 10^−4^ (m^−2^ min^−1^)	3 *10^−10^	9.3 * 10^−6^	120	(15)
LNGS	1,400 m	20nSv / h	3 * 10^−8^	3.78 * 10^−6^	50–120	(16)
CNRS	1,700 m	0.301 or 0.622 (cm^–2^ s^–1^)	4.7 * 10^−9^	5.6 * 10^−6^	15	(17)
DULB-4900	1,800 m	0.02Gy/h	3.0 * 10^−9^	3.8 * 10^−7^	0.85	(18)
CJEM	1,470 m	0.04 μSv/h	NA	NA	148	(19)
CJPL	2,400	NA	2*10^−10^	2.69 * 10^−5^	34–133	(20)

One further contribution to the natural background radiation comes from radon decay products. Radon is a naturally occurring radioactive gas generated by the decay of uranium in rocks and soil, and its decay releases α particles into the air (24, 25). And the hermetic nature of underground mines allows for the significant accumulation of radon, which not only increases the natural background radiation but also has detrimental effects on the health of workers involved in deep underground operations (26, 27). The World Health Organization has reported that radon gas was the second most significant cause of lung cancer after smoking (28). Besides, Rage and Richardson found that the incidence rate of lung cancer among deep underground uranium miners showed a positive correlation with cumulative radon exposure (29, 30). Therefore, it is necessary to focus on the levels and fluctuations of radon gas in the DUGE.

In addition to radiation, the temperature, humidity, and air pressure in the deep underground differ from above ground. Watson et al. (31) measured temperature change with increasing depth in mines and showed that temperatures increase by 3–5°C for every 100 m of depth in the deep underground. Relative humidity and air pressure also rise with increasing depth (32). Our previous studies found that the humidity exceeds 90% at a depth of 600 m in the Erdaogou mine of Jiapigou Minerals Limited Corporation of China National Gold Group Corporation (CJEM) and the China Pingmei Shenma Group (CPSG) mine, which exceeds the acceptable maximum relative humidity for humans (70%) (33, 34). Carbon dioxide and oxygen concentrations in the CJEM and CPSG are similar to above ground, due to appropriate ventilation (33, 34).

## 3. Biological research in the DUGE

Natural background radiation has influenced the evolution of life on Earth for almost 4 billion years (7, 35). The “linear no-threshold” (LNT) model presumes that every exposure to radiation comes with an increased biological risk, and posits that there is no safe level below which harmful effects are not experienced (36, 37). Nevertheless, an expanding body of studies have contradicted the existing model and suggested that the dose–response relationship of cells and tissues under low radiation may not follow a linearity. Examples of phenomena that may deviate from the dose–response relationship include adaptive response and the bystander effects in cell populations (38). The former refers to the phenomenon where cells acquire resistance to the same or even higher doses of radiation after prior exposure to low doses of radiation (39, 40). The bystander effect suggests that cells exposed to low doses of radiation can affect non-irradiated cells through intercellular communication, which results in genomic instability and cellular damage (41). Current understanding of the biological effects of low radiation and LBR is limited. DUL provides a stable long-term chronic LBR environment, which contributes to refining the dose–response relationship of the LNT model and elucidating its underlying mechanisms (13, 42). At present, some DULs are conducting research on the biological effects of LBR in various organisms, including (i) unicellular organisms such as V79 cells, *Shewanella oneidensis*, and *Deinococcus radiodurans* and (ii) multicellular organisms such as *Drosophila melanogaster*, *Caenorhabditis elegans*, and Lake Whitefish. Research is focused on impacts of LBR on the growth, cellular functions, and phenotypes of the model organisms (Table 2).

**Table 2:** The different biological effects of low background radiation on different organisms.

Reference	Laboratory	Depth (m)	Time	Value of radiation dose (LBR vs. CBR; nGy/h)	Cultures	Impact
(8)	SIMP	2,000	A few weeks	Not stated	*Mastigogla*dus I.	Died
(43)	CNRS	200 + 5 cm lead	10 days	11.4 vs. 188.4	*Paramecium tetraurelia*	Growth↓
(9)	WIPP	650	75 h	2 vs. 31	*Deinococcus radiodurans*	Growth↓, HSP70↑
(44)	WIPP	650	72 h	2 vs. 31	*Shewanella oneidensis Deinococcus radiodurans*	Growth↓, KatB↑, SOA0154↑, RecA↑, Dps ↓, GAPDH↓, and DnaK↑
(45)	WIPP	650	77 h	2 vs. 31	*Deinococcus radiodurans*	Growth↓, DnaK↑
(12)	WIPP	605	34 h	0.91 vs. 72.1	*Deinococcus radiodurans*	Growth↓, protein synthesis↓
(13)	Boulby	1,100	7 days	0.5 vs. 112.5	*Bacillus subtilis*, *Escherichia coli*	Growth (−)
(46)	CJEM	1,470	7 days	40 vs. 150	V79	Growth↓, mitochondrial volume↑, hypertrophic ER, ribosomal and spliceosomal protein pathway enrichment, oxidoreductase↓, UQCRH↑, ATP6V1G1↑, and DNA metabolic changes
(47)	CJEM	1,470	4 days	40 vs. 150	FD-LSC-1	Growth↓, ribosomal and spliceosomal protein pathway enrichment, mitochondrial volume↑, hypertrophic ER, UQCRH↑, ATP6V1G1↑, and DNA metabolic changes
(48)	LNGS	1,400	9 months	22 vs. 122	V79	Growth (−), GST (−), SOD↓, and HPRT mutation↑
(49)	WIPP	650	23 days	0.91 (LB) vs. 35 (CB) vs. 72(UB)	V79	Viability↓, transcription-related genes↓
(50)	LNGS	1,400	6 months	3.8 vs. 331.7	TK6	Growth (−), SOD↓, GSH-PX↓, and CAT↓,
(51)	WIPP	640	4 days	0.16 vs. 71	*Shewanella oneidensis*	Growth (−), protein synthesis↓
(52)	LNGS	1,400	9 months	22 vs. 122	*Drosophila melanogaster*	Lifespan↑; fertility↓
(11)	WIPP	650	8 months	15.6 vs. 67.4	*Caenorhabditis elegans* Nematode	Growth↑, egg-laying↑
(10)	SNOLAB	2,000	4 months	11.55 vs. 68.04	Lake Whitefish	Growth↑
(19)	CJEM	1,470	2 days	40 vs. 150	V79	Growth↓, RNA metabolic changes
(53)	LNGS	1,400	Five generations	22 vs. 122	*Drosophila melanogaster*	Chromosome breaks↑
(54)	LNGS	1,400	8 months	22 vs. 122	V79	SOD, CAT, GSH-PX, GSSG-RX, and GST change
(55)	LNGS	1,400	10 months	22.8 vs. 94.7	V79	GSH-Px↓, HPRT mutation↑

SIMP, Simplon pass; CNRS, Center National de la Recherche Scientifique; LNGS, the Gran Sasso National Laboratory; WIPP, Waste Isolation Pilot Plant; SNOLAB, Sudbury Neutrino Observatory Laboratory; CJEML, Laboratory of Jiapigou Minerals Limited Corporation of China National Gold Group Corporation; LBR, low background radiation; CBR, control background radiation; UB, underground background contains K-40; ER, endoplasmic reticulum; UQCRH, ubiquinol cytochrome c reductase hinge; ATP6V1G1, ATPase H+ Transporting V1 Subunit G1; SOD, superoxide dismutase; CAT, catalase; GST, glutathione transferase; GSSG-PX, glutathione peroxidase; GSSG-RX, glutathione reductase; and HPRT, hypoxanthine guanine phosphoribosyl transferase.

### 3.1. LBR affects the growth of organisms

**Figure 2:**
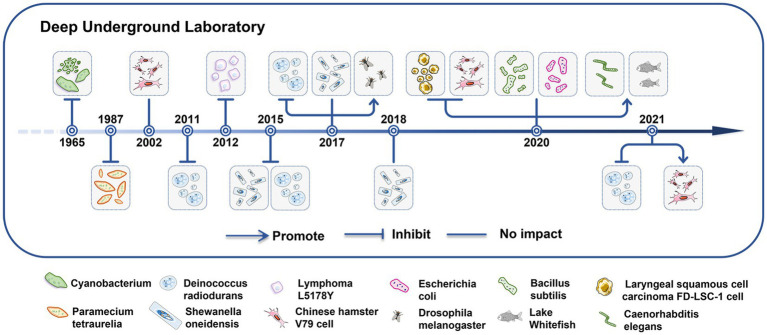
Low background radiation in deep underground laboratories affects the growth of living organisms. The impacts of LBR in the DUGE on the growth patterns of single-celled and multicellular organisms.

In 1964, Eugster indicated that when the cyanobacterium *Mastigocladus laminosus* was cultivated in the Simplon tunnel, it experienced mortality within a matter of week (8) (Figure 2). Planel et al. (43), Smith et al. (9), Kawanishi et al. (56), and Castillo et al. (44, 45) showed significantly reduced growth rates of single-celled organisms cultured in DULs for 3–10 days. In comparison to parallel populations that were cultured above the ground; however, the growth rates of the single-celled organisms cultured in the DULs could be rescued by introduction of an exogenous radiation source that simulated natural background levels (9, 56). Liu confirmed this phenomenon when studying growth rates of mammalian cells for 4–7 days in the CJEM (46, 47). Castillo et al. conducted a research that revealed no impact on the growth rates of *Shewanella oneidensis* when cultured in the Waste Isolation Pilot Plant (WIPP) for 4 days in New Mexico (45, 51). Satta et al. (48) found that V79 cells displayed normal growth rates when cultured in a DUL for 9 months, and Wadsworth et al. (13) elucidated that growth rates of *Bacillus subtilis* and *Escherichia coli* were not affected after 7 days of incubation in a DUL. Castillo et al. (49) discovered that V79 cells cultured for 23 days under LBR in the WIPP had a higher and more variable number of cells and more heterogeneous cell populations compared to cells grown above ground.

In recent years, research in the deep underground has extended to multicellular organisms. Morciano et al. (52) cultured the multicellular organism *Drosophila melanogaster* for 9 months in a DUL and found an increased lifespan but decreased fertility of adult males and females. *Drosophila melanogaster* cultured for five generations showed a stress response and chromosomal damage induced by exposure to LBR. This phenomenon had a cross-generational effect (53). Van Voorhies et al. (11) found that egg-laying rates and larval growth were increased in *Caenorhabditis elegans* exposed to LBR for 8 months compared to those observed under normal background radiation levels. Pirkkanen et al. (10) showed no significant differences in hatching time or survival rates in lake whitefish reared in SNOLAB (2 km underground) for 4 months and on the surface; however, embryos raised underground were larger. The authors speculated that higher radon levels underground (100–150 Bq/m^3^) may potentially account for the notable increase in body size observed in embryos reared within the SNOLAB facility (57).

Although the different organisms maintained in LBR might exhibit different changes in growth, the single-celled organisms presented with growth inhibition in short time might be confirmed. Whereas, a few researches about multicellular cultures and longtime observation for single-celled organisms under LBR environment limited to get a solid conclusion, emphasizing the need for more precise long-term investigations (58).

### 3.2. LBR affects the function of organelles

**Figure 3:**
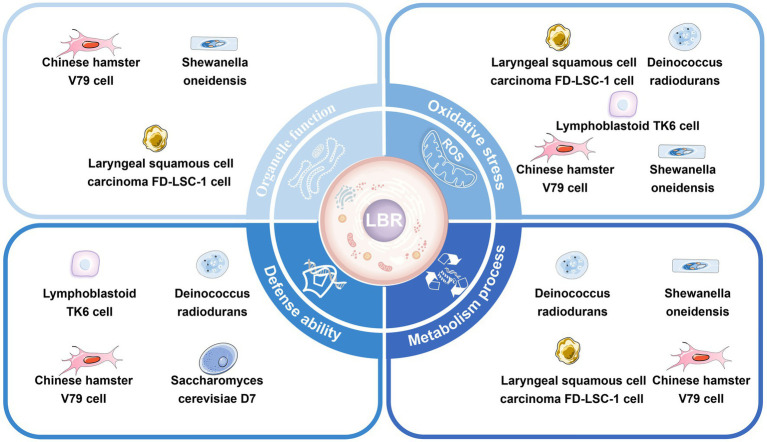
Cellular responses to low background radiation in deep underground laboratories. The low background radiation can induce a stress response and affect the organelles’ function, defense capacity, oxidative stress, and metabolism of cells.

Mitochondria are organelles surrounded by a double-membrane system that provide energy for the activities of eukaryotic cells (59). Mitochondrial reactive oxygen species (ROS) generated during both physiological and pathological conditions have the potential to inflict cellular damage (60, 61). Liu et al. found that mitochondrial volume increased and mitochondria were largely devoid of cristae in V79 cells and FD-LSC-1 cells cultured in a DUL compared to above ground (46, 47) (Figure 3).

As a direct intracellular target of radiation, the endoplasmic reticulum (ER) is highly sensitive to changes in the internal environment (62). Mild ER stress initiates autophagy to restore ER homeostasis and protect cells from damage (63). Liu et al. showed that V79 and FD-LSC-1 cells, when cultivated in a DUL, had hypertrophic ER, obvious Golgi bodies, and downregulation of major ER stress proteins (WFS1, STT3B, CANX, ERP29, and HSPA5) compared to cells cultured above ground (46, 47). Transcriptomics demonstrated that differently expressed (DE) circRNAs in V79 cells cultured in the DUL or above ground were predominantly enriched in genes associated with protein processing in the endoplasmic reticulum (19).

Ribosomes are essential for protein translation and cell proliferation (64). Castillo et al. (51) found a downregulation of ribosomal proteins and tRNA genes in *S. oneidensis* deprived of background radiation compared to controls, suggesting a substantial decrease in protein translation. A similar observation was conducted by Liu et al. who indicated alterations in protein expression associated with the ribosome pathway in V79 and FD-LSC-1 cells cultivated in a DUL compared to above ground. KEGG enrichment analysis of DE proteins showed that ribosome biogenesis and ribosomal proteins (RPS6, RPS14, RPS16, RPL8, RPL23, RPL3, and RPS18) played a key role in regulating cell growth in cells cultured in the DUL (46, 47).

Spliceosomes are essential organelles that play a pivotal role in cell growth by regulating gene expression (65). Liu et al. found that DE proteins between V79 and FD-LSC-1 cells and DE LncRNAs between V79 cells cultured in a DUL or above ground were significantly enriched in the spliceosome pathway (19, 46, 47).

Together, these data imply that mitochondria, the ER, ribosomes, and the spliceosome are involved in the stress and adaptive responses in cells exposed to LBR. The mechanism underlying the role of these organelles in cellular adaptation in a DUGE require further research.

### 3.3. LBR causes phenotypic alterations in organisms

#### 3.3.1. Oxidative stress

Most biological research on LBR conducted in DULs has focused on cellular oxidative stress. Antonelli et al. discovered changes in antioxidant enzyme activities [superoxide dismutase (SOD), catalase (CAT), glutathione peroxidase, reductase, and transferase (GSH-PX, GSSG-RX, and GST)] in V79 cells grown in the LNGS (54). Satta et al. (48) showed SOD activity was lower in V79 cells grown in LBR, as compared to those cultured in a standard environment for 9 months, while there was no difference in GST activity. Carbone et al. (50) found that SOD, GSH-PX, and CATase were reduced in human lymphoblastoid TK6 cells cultured in reduced compared to reference environmental radiation conditions. Smith et al. (9) reported an increase in the expression of the heat shock protein 70 (HSP70) in primary human lung fibroblasts and bronchial epithelial cells cultured in reduced radiation levels compared to background. Fratini et al. found a decrease in cellular GSH-Px activity in V79 cells cultured for 10 months in LBR, in comparison to cells cultured in a reference environment. This biological response did not revert in cells brought back to the reference-radiation environment for 6 months (55). Castillo et al. demonstrated that the oxidative stress-related genes katB and SOA0154 and the DNA damage-related gene recA were upregulated in *S. oneidensis*, while dps (DNA protection against ROS) and gapdH (ATP synthesis) genes were downregulated in *Deinococcus radiodurans* incubated in the WIPP for 76 h compared to background radiation control (44).

Liu et al. performed transcriptomic and proteomic analyses of V79 and FD-LSC-1 cells cultured in a DUL compared to above ground. Withdrawal of background radiation downregulated proteins enriched in oxidoreductase activity and the redox process (46) and upregulated ubiquinol cytochrome c reductase hinge (UQCRH) and ATPase H+ Transporting V1 Subunit G1 (ATP6V1G1) (46, 47). Transcriptomic analysis showed that the DE mRNAs between V79 cells grown in the DUL or above ground were mainly enriched in redox activity and redox processes, and the DE mRNAs of FD-LSC-1 cells grown in a DUL or above ground were also enriched in SOD-related entities (19).

Together, these data imply that LBR in the DUGE could induce oxidative stress in cells. However, the core mechanism and target factors remain to be elucidated.

#### 3.3.2. Defense against external factors

Satta et al. (66) found a higher frequency of radiomimetic induced recombination in *S cerevisiae* D7 exposed to recombinogenic doses of methyl methanesulfonatre grown in LBR compared to a standard environment. A subsequent study showed that V79 cells grown in LBR for 9 months exhibited enhanced susceptibility to cycloheximide-induced apoptosis and had an increased frequency of mutations in the hypoxanthine guanine phosphoribosyl transferase (hprt) gene induced by γ-ray exposure compared to cells cultivated in normal background radiation (48). Similarly, Fratini et al. and Antonelli et al. observed an increased rate of spontaneous hprt mutations in V79 cells cultured in a LBR environment as opposed to a standard radiation (54, 55). Carbone et al. (50, 67) demonstrated that the degree of DNA damage and repair and management of ROS balance was different in TK6 cells exposed to 2Gy X-ray irradiation in a DUL as opposed to a standard environment for 6 months. Castillo et al. (44, 45) showed upregulation of dnaK *in D. radiodurans* cultured in LBR compared to background radiation.

Together, these findings imply that LBR may increase the frequency of gene recombination and susceptibility of cells to damaging factors.

#### 3.3.3. Metabolic responses

Metabolic processes involving DNA, RNA, and proteins control cell growth and proliferation. Metabolic processes in organisms exposed to LBR are altered. Castillo et al. found that protein synthesis was inhibited, reflected by a reduced number of ribosomes and downregulation of tRNA, and transcription of HSP genes was reduced, in *D. radiodurans* exposed to LBR environment as opposed to a reference environment (12, 51). In another study, Castillo et al. (49) used GO analysis to show that transcription-related genes were downregulated in V79 cells cultured in a DUL compared to above ground. Duan et al. (19) and Liu et al. (68) revealed an altered RNA profile compared to control, which may have negative impacts on metabolic processes, in V79 and FD-LSC-1 cells grown in LBR in the DUGE. Liu et al. performed proteomic analysis of V79 and FD-LSC-1 cells cultivated in a DUL compared to above ground and demonstrated modifications in DNA replication, transcription, translation, and protein modification, which may be related to a LBR stress response (46, 47). Liu et al. (68) also showed that the TGF-β and Hippo signaling pathway and the cell proliferation-related genes SMAD, SMAD7, CDH1, EGR1, and BMP2 played key roles in the transcriptional regulation of FD-LSC-1 cells grown in an LBR environment.

Together, these data imply that living systems can perceive LBR in the DUGE and generate stress responses that result in changes to metabolic processes involving protein synthesis.

## 4. Advancing deep underground medical research

With the prospect of a growing number of individuals inhabiting and working in the deep underground space, some research been dedicated to exploring the effect of the DUGE on human pathology, psychology, and physiology.

In relation to human pathology, Strzemecka et al. surveyed 700 people working underground in the Bogdanka coal mine at a depth of 1,100 m. They revealed that 50% of the miners perceived the microclimate in the mine, including high humidity and high temperatures, threatened their health (32). Meshi et al. reported that 78.4% of underground miners of the Mara gold mine in Tanzania suffered from moderate heat-related illness, manifested by high body temperature and hot and dry skin. The Mara gold mine is located in the tropics at a depth of 500 m and has an ambient temperature of 28.4°C and relative humidity over 58.2% (69). Liu et al. described the subjective perceptions and mental state of employees working in the CJEM (1,470 m) and CPSG (1,500 m). Moisture and heat were the most commonly perceived adverse factors in the deep underground spaces (33, 34).

In relation to human psychology, it is known that employees working for long periods in shallow coal mines suffer depression and anxiety (70–72). Liu et al. investigated the mental state of 496 miners who worked for long periods of time at a depth below 600 m in the CJEM and CPSG. Workers at both mines reported being easily fatigued and having trouble sleeping and waking up early in the morning (33, 34). In relation to human physiology, urine metabolomics analysis indicates that the DUGE induces specific changes in the metabolism of amino acids (L-phenylalanine, L-tyrosine, and L-glutamine) in humans (73).

## 5. Conclusion and perspective research in the deep underground

The utilization of deep underground resources and the development of deep underground spaces have become a major strategy for many developed countries. However, research suggests that the unique DUGE could induce a stress response in living organisms and be harmful to humans. Currently, knowledge about the DUGE is limited, making it difficult to support further development. In future, it is necessary to explore the biological impacts of LBR in the DUGE on living systems. DULs provide a unique platform where the biological effects of various components of radiation (radon gas, γ, etc.) and dose responses can be explored. Some data imply that mitochondria, the ER, ribosomes, and the spliceosome are responsible for a stress response in cells cultured in a LBR environment. However, the interconnections and feedback control between organelles and underlying molecular mechanisms remain unclear. Interestingly, LBR in the DUGE has an inhibitory effect on the proliferation of cancer cells, especially radiation-sensitive cancer cells, providing a potential new perspective for cancer control.

Some studies have shown that the DUGE affects human pathology, psychology, and physiology. Therefore, a comprehensive qualitative and quantitative assessment is imperative to accurately characterize the factors in the DUGE (rock, humidity, temperature, illumination etc.) and their potential advantageous or detrimental impacts on humans. Findings may inform strategies that facilitate human adaptation and habituation to the DUGE. The long-term consequences of exposure to factors in the DUGE in humans must be monitored, and cross-disciplinary research between DUGM and various clinical disciplines is required to support sustained development and utilization of the deep underground space.

## Author contributions

JWe, JC, and CL collated and analyzed the literature. ZH, JZ, and MG are helpful for completing diagrams and writing papers. WL, JWu, and HX wrote sections of the manuscript. All authors contributed to the article and approved the submitted version.

## Funding

The authors would like to express their gratitude for the support provided by the Special funds for deep underground medical research of West China hospital, Sichuan University (grant no. YB2018002) and 1.3.5 project for disciplines of excellence, West China Hospital, Sichuan University (grant no. ZYJC21048 and 18016), Foundation of Sichuan Provincial Science and Technology Program (grant no. 2022JDR0091), 2020 Cooperation project for Sichuan University and Yibin Municipal People’s Government (grant no. 2020CDYB-35), and cooperation project for Academician & Experts Workstation (grant no. HXYS20001).

## Conflict of interest

The authors declare that the research was conducted in the absence of any commercial or financial relationships that could be construed as a potential conflict of interest.

## Publisher’s note

All claims expressed in this article are solely those of the authors and do not necessarily represent those of their affiliated organizations, or those of the publisher, the editors and the reviewers. Any product that may be evaluated in this article, or claim that may be made by its manufacturer, is not guaranteed or endorsed by the publisher.

## References

[1] Ranjith PG Zhao J Ju M De Silva RVS Rathnaweera TD Bandara AKMS. Opportunities and challenges in deep mining: a brief review. Engineering. (2017) 3:546–51. doi: 10.1016/j.Eng.2017.04.024

[2] Yu H Gao Y Zhou R. Oxidative stress from exposure to the underground space environment. Front Public Health. (2020) 8:579634. doi: 10.3389/fpubh.2020.579634, PMID: 33194980 PMC7609794

[3] Xie H. Research framework and anticipated results of deep rock mechanics and mining theory. Adv Eng Sci. (2017) 49:1–16. doi: 10.15961/j.jsuese.201700025

[4] Huang B Zhang N Jing H Han J Meng B Li N. Large deformation theory of rheology and structural instability of the surrounding rock in deep mining roadway. J China Coal Soc. (2020) 45:911–26. doi: 10.13225/j.cnki.jccs.SJ19.1451

[5] Xie HP Liu JF Gao MZ Wan XH Liu SX Zou J. The research advancement and conception of the deep-underground medicine. Sichuan Da Xue Xue Bao Yi Xue Ban. (2018) 49:163–8. doi: 10.13225/j.cnki.jccs.FQ21.1564 29737053

[6] Laubenstein M Lawson I. Low background radiation detection techniques and mitigation of radioactive backgrounds. Front Phys. (2020) 8:577734. doi: 10.3389/fphy.2020.577734

[7] UNSCEA SOURCES. Sources and Effects of Ionizing Radiation, UNSCEAR 2000 Report to the General Assembly, With Scientific Annexes. New York, NY: United Nations. (2000).

[8] Eugster JG. Subradiation experiments concerning the concept of the natural radiation environment. Aerosp Med. (1964) 35:524–6. 14154168

[9] Smith GB Grof Y Navarrette A Guilmette RA. Exploring biological effects of low level radiation from the other side of background. Health Phys. (2011) 100:263–5. doi: 10.1097/HP.0b013e318208cd44, PMID: 21595063

[10] Pirkkanen J Zarnke AM Laframboise T Lees SJ Tai TC Boreham DR. A research environment 2 km deep-underground impacts embryonic development in lake whitefish (*Coregonus clupeaformis*). Front Earth Sci. (2020) 8:327. doi: 10.3389/feart.2020.00327

[11] Van Voorhies WA Castillo HA Thawng CN Smith GB. The phenotypic and transcriptomic response of the caenorhabditis elegans nematode to background and below-background radiation levels. Front Public Health. (2020) 8:581796. doi: 10.3389/fpubh.2020.581796, PMID: 33178665 PMC7596186

[12] Castillo H Li X Smith GB. *Deinococcus radiodurans* UWO298 dependence on background radiation for optimal growth. Front Genet. (2021) 12:644292. doi: 10.3389/fgene.2021.644292, PMID: 34025716 PMC8136434

[13] Wadsworth J Cockell CS Murphy AS Nilima A Paling S Meehan E. There’s plenty of room at the bottom: low radiation as a biological extreme. Front Astronomy Space Sci. (2020) 7:50. doi: 10.3389/fspas.2020.00050

[14] Malczewski D Kisiel J Dorda J. Gamma background measurements in the Boulby underground laboratory. J Radioanal Nucl Chem. (2013) 298:1483–9. doi: 10.1007/s10967-013-2540-9, PMID: 26224946 PMC4513908

[15] Kennedy KJ LeBlanc A Pirkkanen J Thome C Tai TC LeClair R. Dosimetric characterisation of a sub-natural background radiation environment for radiobiology investigations. Radiat Prot Dosim. (2021) 195:114–23. doi: 10.1093/rpd/ncab120, PMID: 34402520

[16] Esposito G Anello P Ampollini M Bortolin E De Angelis C D'Imperio G. Underground radiobiology: a perspective at gran Sasso National Laboratory. Front Public Health. (2020) 8:611146. doi: 10.3389/fpubh.2020.611146, PMID: 33365298 PMC7750398

[17] Malczewski D Kisiel J Dorda J. Gamma background measurements in the Laboratoire Souterrain de Modane. J Radioanal Nucl Chem. (2012) 292:751–6. doi: 10.1007/s10967-011-1497-9 26224920 PMC4514586

[18] Zarubin M Gangapshev A Gavriljuk Y Kazalov V Kravchenko E. First transcriptome profiling of *D. melanogaster* after development in a deep underground low radiation background laboratory. PLoS One. (2021) 16:e0255066. doi: 10.1371/journal.pone.0255066 34351964 PMC8341612

[19] Duan L Jiang H Liu J Liu Y Ma T Xie Y. Whole transcriptome analysis revealed a stress response to deep underground environment conditions in chinese hamster V79 lung fibroblast cells. Front Genet. (2021) 12:698046. doi: 10.3389/fgene.2021.698046, PMID: 34603371 PMC8481809

[20] Cheng JP Kang KJ Li JM Li J Li YJ Yue Q. The China jinping underground laboratory and its early science. Annu Rev. (2017) 67:231–51. doi: 10.1146/annurev-nucl-102115-044842

[21] Morciano P Cipressa F Porrazzo A Esposito G Tabocchini MA Cenci G. Fruit flies provide new insights in low-radiation background biology at the INFN underground gran Sasso National Laboratory (LNGS). Radiat Res. (2018) 190:217–25. doi: 10.1667/rr15083.1, PMID: 29863430

[22] Zarubin MP Kuldoshina OA Kravchenko EV. Biological effects of low background radiation: prospects for future research in the low-background laboratory DULB-4900 of Baksan neutrino observatory INR RAS. Phys Part Nucl. (2021) 52:19–30. doi: 10.1134/S1063779621010056

[23] Bacioiu I. Equivalent dose rate by muons to the human body. Radiat Prot Dosim. (2011) 147:380–5. doi: 10.1093/rpd/ncq460, PMID: 21147787

[24] Gil-Oncina S Valdes-Abellan J Pla C Benavente D. Estimation of the radon risk under different european climates and soil textures. Front Public Health. (2022) 10:794557. doi: 10.3389/fpubh.2022.794557, PMID: 35252086 PMC8892385

[25] Stekl I Hulka J Mamedov F Fojtik P Cermakova E Jilek K. Low radon cleanroom for underground laboratories. Front Public Health. (2020) 8:589891. doi: 10.3389/fpubh.2020.589891, PMID: 33604322 PMC7884809

[26] Elio J Crowley Q Scanlon R Hodgson J Zgaga L. Estimation of residential radon exposure and definition of radon priority areas based on expected lung cancer incidence. Environ Int. (2018) 114:69–76. doi: 10.1016/j.envint.2018.02.025, PMID: 29486412

[27] UNSCEAR Sources, Effects and Risks of Ionizing Radiation, UNSCEAR 2019 Report to the General Assembly, With Scientific Annexes. United Nations, New York, NY. (2020).

[28] WHO Handbook on Indoor Radon A Public Health Perspective. WHO Guidelines Approved by the Guidelines Review Committee. Geneva. (2009). 23762967

[29] Rage E Richardson DB Demers PA Do M Fenske N Kreuzer M. PUMA—pooled uranium miners analysis: cohort profile. Occup Environ Med. (2020) 77:194–200. doi: 10.1136/oemed-2019-105981, PMID: 32005674 PMC8663280

[30] Richardson DB Rage E Demers PA Do MT Fenske N Deffner V. Lung cancer and radon: pooled analysis of uranium miners hired in 1960 or later. Environ Health Perspect. (2022) 130:57010. doi: 10.1289/EHP10669, PMID: 35604341 PMC9126132

[31] Watson TL. Underground temperatures. Science. (1911) 33:828–31. doi: 10.1126/science.33.856.828 17795878

[32] Strzemecka J Gozdziewska M Skrodziuk J Galinska EM Lachowski S. Factors of work environment hazardous for health in opinions of employees working underground in the 'Bogdanka' coal mine. Ann Agric Environ Med. (2019) 26:409–14. doi: 10.26444/aaem/106224, PMID: 31559795

[33] Liu J Liu Y Ma T Gao M Zhang R Wu J. Subjective perceptions and psychological distress associated with the deep underground: a cross-sectional study in a deep gold mine in China. Medicine. (2019) 98:e15571. doi: 10.1097/md.0000000000015571, PMID: 31145277 PMC6708914

[34] Xie H Liu J Gao M Liu Y Ma T Lu Y. Physical symptoms and mental health status in deep underground miners: a cross-sectional study. Medicine. (2020) 99:e19294. doi: 10.1097/md.0000000000019294, PMID: 32118742 PMC7478699

[35] Belli M Indovina L. The response of living organisms to low radiation environment and its implications in radiation protection. Front Public Health. (2020) 8:601711. doi: 10.3389/fpubh.2020.601711, PMID: 33384980 PMC7770185

[36] UNSCEAR. Biological mechanisms of radiation actions at low doses. New York, NY: United Nations (2012).

[37] Liu J Ma T Liu Y Zou J Gao M Zhang R. History, advancements, and perspective of biological research in deep-underground laboratories: a brief review. Environ Int. (2018) 120:207–14. doi: 10.1016/j.envint.2018.07.031 30098554

[38] Adelstein SJ. Biologic responses to low doses of ionizing radiation: adaptive response versus bystander effect. J Nucl Med. (2003) 44:125. doi: 10.1067/mnc.2003.18 12515886

[39] Mitchel RE. Adaption by low dose radiation exposure: a look at scope and limitations for radioprotection. Dose-Response. (2015) 1:1–7. doi: 10.2203/dose-response.14-025.Mitchel PMC4674178 26672725

[40] Yu X Wang H Wang P Chen BP Wang Y. The Ku-dependent non-homologous end-joining pathway contributes to low-dose radiation-stimulated cell survival. J Cell Physiol. (2011) 226:369–74. doi: 10.1002/jcp.22342, PMID: 20665702 PMC4079012

[41] Hall EJ. The bystander effect. Health Phys. (2003) 85:31–5. doi: 10.1097/00004032-200307000-00008 12852468

[42] UNSCEAR Sources, Effects and Risks of Ionizing Radiation, UNSCEAR 2017 Report to the General Assembly, With Scientific Annexes. New York, NY: United Nations. (2018).

[43] Planel H Soleilhavoup JP Tixador R Richoilley G Conter A Croute F. Influence on cell proliferation of background radiation or exposure to very low, chronic gamma radiation. Health Phys. (1987) 52:571–8. doi: 10.1097/00004032-198705000-00007, PMID: 3106264

[44] Castillo H Schoderbek D Dulal S Escobar G Wood J Nelson R. Stress induction in the bacteria Shewanella oneidensis and *Deinococcus radiodurans* in response to below-background ionizing radiation. Int J Radiat Biol. (2015) 91:749–56. doi: 10.3109/09553002.2015.1062571, PMID: 26073528

[45] Castillo H Smith GB. Below-background ionizing radiation as an environmental cue for bacteria. Front Microbiol. (2017) 8:177. doi: 10.3389/fmicb.2017.00177

[46] Liu J Ma T Gao M Liu Y Liu J Wang S. Proteomics provides insights into the inhibition of Chinese hamster V79 cell proliferation in the deep underground environment. Sci Rep. (2020a) 10:14921. doi: 10.1038/s41598-020-71154-z, PMID: 32913333 PMC7483447

[47] Liu J Ma T Gao M Liu Y Liu J Wang S. Proteomic characterization of proliferation inhibition of well-differentiated laryngeal squamous cell carcinoma cells under below-background radiation in a deep underground environment. Front Public Health. (2020b) 8:584964. doi: 10.3389/fpubh.2020.584964, PMID: 33194991 PMC7661695

[48] Satta L Antonelli F Belli M Sapora O Simone G Sorrentino E. Influence of a low background radiation environment on biochemical and biological responses in V79 cells. Radiat Environ Biophys. (2002) 41:217–24. doi: 10.1007/s00411-002-0159-2, PMID: 12373331

[49] Castillo H Winder J Smith G. Chinese hamster V79 cells' dependence on background ionizing radiation for optimal growth. Radiat Environ Biophys. (2022) 61:49–57. doi: 10.1007/s00411-021-00951-5, PMID: 34751828

[50] Carbone MC Pinto M Antonelli F Amicarelli F Balata M Belli M. The cosmic silence experiment: on the putative adaptive role of environmental ionizing radiation. Radiat Environ Biophys. (2009) 48:189–96. doi: 10.1007/s00411-008-0208-6 19169701

[51] Castillo H Li X Schilkey F Smith GB. Transcriptome analysis reveals a stress response of *Shewanella oneidensis* deprived of background levels of ionizing radiation. PloS one. (2018) 13:e0196472. doi: 10.1371/journal.pone.0196472, PMID: 29768440 PMC5955497

[52] Morciano P Iorio R Iovino D Cipressa F Esposito G Porrazzo A. Effects of reduced natural background radiation on *Drosophila melanogaster* growth and development as revealed by the FLYINGLOW program. J Cell Physiol. (2018) 233:23–9. doi: 10.1002/jcp.25889, PMID: 28262946

[53] Porrazzo A Esposito G Grifoni D Cenci G Morciano P Tabocchini MA. Reduced environmental dose rates are responsible for the increased susceptibility to radiation-induced DNA damage in larval neuroblasts of drosophila grown inside the LNGS underground laboratory. Int J Mol Sci. (2022) 23:5472. doi: 10.3390/ijms23105472 35628279 PMC9143493

[54] Antonelli F Belli M Sapora O Simone G Sorrentino E Tabocchini MA. Radiation biophysics at the gran Sasso laboratory: influence of a low background radiation environment on the adpative response of living cells. Nuclear Phys B Proc Suppl. (2000) 87:508–9. doi: 10.1016/S0920-5632(00)00735-0

[55] Fratini E Carbone C Capece D Esposito G Simone G Tabocchini MA. Low-radiation environment affects the development of protection mechanisms in V79 cells. Radiat Environ Biophys. (2015) 54:183–94. doi: 10.1007/s00411-015-0587-4 25636513

[56] Kawanishi M Okuyama K Shiraishi K Matsuda Y Taniguchi R Shiomi N. Growth retardation of paramecium and mouse cells by shielding them from background radiation. J Radiat Res. (2012) 53:404–10. doi: 10.1269/jrr.11145, PMID: 22739010

[57] Thome C Mitz C Hulley EN Somers CM Manzon RG Wilson JY. Initial characterization of the growth stimulation and heat-shock-induced adaptive response in developing lake whitefish embryos after ionizing radiation exposure. Radiat Res. (2017) 188:475–85. doi: 10.1667/RR14574.1, PMID: 28737450

[58] Daly MJ Gaidamakova EK Matrosova VY Vasilenko A Zhai M Venkateswaran A. Accumulation of Mn (II) in *Deinococcus radiodurans* facilitates gamma-radiation resistance. Science. (2004) 306:1025–8. doi: 10.1126/science.1103185, PMID: 15459345

[59] Lu Z Wang S Ji C Li F Cong M Shan X. iTRAQ-based proteomic analysis on the mitochondrial responses in gill tissues of juvenile olive flounder *Paralichthys olivaceus* exposed to cadmium. Environ Pollut. (2020) 257:113591. doi: 10.1016/j.envpol.2019.113591 31744679

[60] Napolitano G Fasciolo G Venditti P. Mitochondrial management of reactive oxygen species. Antioxidants. (2021) 10:1824. doi: 10.3390/antiox10111824, PMID: 34829696 PMC8614740

[61] Wilson DF. Oxidative phosphorylation: regulation and role in cellular and tissue metabolism. J Physiol. (2017) 595:7023–38. doi: 10.1113/JP273839, PMID: 29023737 PMC5709332

[62] Wang JS Wang HJ Qian HL. Biological effects of radiation on cancer cells. Mil Med Res. (2018) 5:20. doi: 10.1186/s40779-018-0167-4, PMID: 29958545 PMC6026344

[63] Scheper W Nijholt DA Hoozemans JJ. The unfolded protein response and proteostasis in Alzheimer disease: preferential activation of autophagy by endoplasmic reticulum stress. Autophagy. (2011) 7:910–1. doi: 10.4161/auto.7.8.15761 21494086 PMC3149694

[64] Zhou X Liao WJ Liao JM Liao P Lu H. Ribosomal proteins: functions beyond the ribosome. J Mol Cell Biol. (2015) 7:92–104. doi: 10.1093/jmcb/mjv014, PMID: 25735597 PMC4481666

[65] Will CL Luhrmann R. Spliceosome structure and function. Cold Spring Harb Perspect Biol. (2011) 3:a003707. doi: 10.1101/cshperspect.a003707, PMID: 21441581 PMC3119917

[66] Satta L Augusti-Tocco G Ceccarelli R Esposito A Fiore M Paggi P. Low environmental radiation background impairs biological defence of the yeast *Saccharomyces cerevisiae* to chemical radiomimetic agents. Mutat Res. (1995) 347:129–33. doi: 10.1016/0165-7992(95)00031-3, PMID: 7565903

[67] Carbone MC Pinto M Antonelli F Balata M Satta L. Effects of deprivation of background environmental radiation on cultured human cells. Nuovo Cimento Soc Ital Fisica Sezione B. (2010) 125:469–77. doi: 10.1393/ncb/i2010-10889-y

[68] Liu Y Gao Y Cheng J Ma T Xie Y Wen Q. Transcriptome analysis of well-differentiated laryngeal squamous cell carcinoma cells in below-background environment. Ann Transl Med. (2022) 10:824. doi: 10.21037/atm-22-2997 36035002 PMC9403920

[69] Meshi EB Kishinhi SS Mamuya SH Rusibamayila MG. Thermal exposure and heat illness symptoms among workers in mara gold mine, Tanzania. Ann Glob Health. (2018) 84:360–8. doi: 10.29024/aogh.2318, PMID: 30835389 PMC6748306

[70] Liu L Wang L Chen J. Prevalence and associated factors of depressive symptoms among Chinese underground coal miners. Biomed Res Int. (2014) 2014:987305. doi: 10.1155/2014/987305 24707503 PMC3953506

[71] McLean KN. Mental health and well-being in resident mine workers: out of the fly-in fly-out box. Aust J Rural Health. (2012) 20:126–30. doi: 10.1111/j.1440-1584.2012.01267.x, PMID: 22620476

[72] Hristov ZI. Psychoemotional stress of employees and workers in the public and real sectors of national economy in Bulgaria. Folia Med. (2009) 51:58–67. 19670542

[73] Wen Q Zhou J Sun X Ma T Liu Y Xie Y. Urine metabolomics analysis of sleep quality in deep-underground miners: a pilot study. Front Public Health. (2022) 10:969113. doi: 10.3389/fpubh.2022.969113, PMID: 36062104 PMC9437423

